# Evaluation of Combined LED-Fluorescence Microscopy and Bleach Sedimentation for Diagnosis of Tuberculosis at Peripheral Health Service Level

**DOI:** 10.1371/journal.pone.0020175

**Published:** 2011-05-31

**Authors:** Maryline Bonnet, Laramie Gagnidze, Philippe J. Guerin, Laurence Bonte, Andrew Ramsay, Willie Githui, Francis Varaine

**Affiliations:** 1 Epicentre, Paris, France; 2 Médecins Sans Frontières, Paris, France; 3 UNICEF/UNDP/World Bank/WHO Special Programme for Research and Training in Tropical Diseases (TDR), Geneva, Switzerland; 4 Centre for Respiratory Diseases Research, Kenya Medical Research Institute, Nairobi, Kenya; San Francisco General Hospital, University of California San Francisco, United States of America

## Abstract

**Background:**

Sputum microscopy is the only diagnostic for tuberculosis (TB) available at peripheral levels of health service in resource-poor countries. Its sensitivity is reduced in high HIV-prevalence settings. Sodium hypochlorite (NaOCl) specimen sedimentation prior microscopy and light-emitting diode (LED)-fluorescence microscopy (FM) can individually improve performance of microscopy. This study aimed to evaluate the performance of combined LED-FM and NaOCl sputum sedimentation for TB detection at peripheral level of health services.

**Methods:**

A prospective study was conducted in an urban health clinic in Nairobi, Kenya. Three sputum specimens were collected over 2 days from consecutive TB suspects. Smears were prepared and stained with auramine O and Ziehl-Neelsen (ZN) methods. Bleach (3.5%) was added to the remaining specimen before overnight sedimentation at room temperature. Auramine O staining was performed on smears of sediment. A 4^th^ specimen was collected for TB culture. Auramine smears were read under the same microscope as used for ZN smears, but equipped with the LED FluoLED™ fluorescence illuminator.

**Results:**

497 patients were included, and 1394 specimens collected. The yield of positive specimen was significantly increased after NaOCl sedimentation (24.9%) compared to direct LED-FM (20.6%) and direct ZN (20.3%). In detecting smear-positive patients, sensitivity was 78.5% for LED-FM after NaOCl sedimentation compared to 73.2% and 72.0% for direct LED-FM (*P* = 0.06) and direct ZN (*P* = 0.06), respectively. Specificity was 87.8% for LED-FM after NaOCl sedimentation compared to 96.7% and 95.9% for direct LED-FM (*P*<0.01) and direct ZN (*P*<0.01), respectively. Inter-reading agreement (kappa = 0.7) and technicians' acceptability were good.

**Conclusion:**

NaOCl sedimentation did not improve the performance of LED-FM in the diagnosis of pulmonary TB at peripheral health service level.

## Introduction

Despite renewed efforts to control the epidemic, tuberculosis (TB) remains a public health emergency predominantly affecting the poorest countries of the world, especially in high HIV-prevalence regions [1]. Serial sputum smear microscopy is the only available test to confirm TB that is suitable and affordable for implementation at lower levels of the health service. Unfortunately, TB microscopy is associated with low and variable sensitivity, particularly in high HIV-prevalence settings [2].

Extensive laboratory infrastructure strengthening, as well as development of more sensitive and rapid TB diagnostics suitable for primary health care settings, is urgently needed but not expected in the short term [3], [4]. Recognizing this, a number of research groups have aimed to improve the performance of smear microscopy through new technology and service delivery approaches [5]–[10]. The WHO Strategic and Technical Advisory Group (STAG) for TB recommended that fluorescence microscopy (FM) using light-emitting diode (LED) be phased in as an alternative for conventional Ziehl-Neelsen (ZN) microscopy [11]. In addition to 10% improvement of sensitivity when using conventional FM compared to ZN microscopy with comparable specificity, recent advances of simple LED-based FM systems can allow implementation of FM in low levels of health service [6], [11]. A number of LED-FM microscopes and adaptors that can convert existing light microscopes to LED-FM have been evaluated and are on the market [12]–[17].

Sputum processing methods using household bleach (sodium hypochlorite [NaOCl]) combined with either centrifugation or sedimentation before smear microscopy were also identified as promising approaches to improve performance of smear microscopy [3], [18]. Among these approaches, overnight NaOCl sedimentation has been considered suitable for lower levels of health services [18]. Compared to approaches involving centrifugation, this method is cheaper, safer, and does not necessarily require electrical power. In a recent systematic review (2010) of studies evaluating the accuracy of different processing methods compared to direct ZN microscopy, and using TB culture as reference standard, bleach sedimentation was 9% more sensitive than direct microscopy (95% CI: 4–14%, *P* = 0.001) [19]. Our group in Kenya reported a 23% increase in case-detection yield [20]. All published studies of the bleach sedimentation method to date stained bleach-processed smears using ZN.

Taking into consideration the potential for future wide-scale implementation of the LED-FM, this study aimed to assess if overnight bleach sedimentation would enhance the performance of LED-FM in detecting TB, when used in a high HIV-prevalence country at a peripheral health center. Field-level operational aspects of using the bleach sedimentation method in combination with LED-FM were also assessed.

## Methods

Results comparing the performance of direct ZN microscopy and direct LED-FM from the same study population have been previously published [20]. The study was conducted in an urban outpatient clinic supported by Médecins Sans Frontières in Mathare, a slum area of Nairobi, Kenya. Patients and specimen collection were detailed in the previous publication [20]. Consecutive patients over 15 years of age presenting with cough longer than 2 weeks were eligible for the study. Three sputum specimens were collected over 2 consecutive days [20].

Two smears were made of each specimen for direct smear microscopy. One was stained using the hot ZN method (1% filtered carbol-fuchsin, 0.1% methylene blue) and the other stained for 15 minutes using the auramine O method (0.5% solution, Merck, Darmstadt, Germany) for FM. Auramine smears were counterstained with potassium permanganate (0.5% solution, Merck, Darmstadt, Germany) for 60 seconds. The remainder of the specimen was transferred to a 15-mL disposable plastic conical tube with an equal volume of neat 3.5% commercial bleach (Jik bleach regular, active ingredient: sodium hypochlorite. 3.5% m/v when packed, Reckitt Benckiser, East Africa Ltd, Nairobi, Kenya) and left on the bench for overnight sedimentation, as previously described [21]. After sedimentation, the supernatant was poured off and the sediment mixed with the remaining fluid. One to two drops were transferred to a slide using a sterile glass pipette. A smear was made and stained using the auramine O method for FM.

ZN slides were examined by bright-field microscopy (magnification, 1000X), and auramine slides were examined under the same microscope equipped with LED FluoLED™ fluorescence illuminator (Fraen Corporation Srl, Cusago, Italy) under 400X [22]. To avoid the problem of fading of the auramine-stained slides, all were examined within 24 hours after staining. Laboratory technicians reading the slides were blind to the results of previous smears from the same patient [20]. The technicians did not have prior experience with LED-FM and received 2 weeks of training of FM reading before the study. The exact number of acid-fast bacilli (AFB) observed in one length of each smear was recorded [20]. The time required to stain daily batches of auramine slides (direct and after NaOCl sedimentation) and to read individual slide was measured.

All study slides were kept in opaque slide boxes in the laboratory with monitoring of temperature. On a monthly basis, as part of internal quality control (IQC), the study laboratory supervisor read blindly a random sample of 50–100% positive ZN smears and 10–20% negative ZN smears. Because LED-FM slides should be read within 24 hours to avoid problems with fading, once a week the study laboratory supervisor re-checked all FM slides of the day, unannounced and blindly. External quality assessment of smear reading was performed for ZN smears at the end of the study on a random selection of 120 slides at the Kenya Medical Research Institute (KEMRI; Nairobi, Kenya). The KEMRI laboratory has been externally quality controlled since 1997 by the Mycobacterium Reference Unit of the Clinical Research Centre, Barts and The London School of Medicine and Dentistry, Queen Mary, University of London, UK; and since 2006 by the Mycobacteriology Unit at the Institute of Tropical Medicine, Antwerp, Belgium. In accordance with guidance from the IUATLD Working Group on Sputum Smear Microscopy, positive LED-FM slides were not confirmed by restaining with ZN [23].

A random selection of 200 direct and concentrated auramine-stained smears was read blindly by a second reader on the same day to measure the inter-reader reliability. The laboratory supervisor masked the identification of the slide to ensure the blindness of the reading.

The patient was asked to produce a fourth early-morning sputum specimen at home on the third day and to bring this the same day to the laboratory when s/he came to collect microscopy results. Specimens were stored at 2–4°C and sent twice weekly to the KEMRI laboratory where the *Mycobacterium tuberculosis* (MTB) culture on Lowenstein Jensen (LJ) medium was performed as reference standard. Specimens were decontaminated using N-acetyl-L-cysteine/sodium hydroxide NALC/NaOH, followed by neutralization with phosphate buffer, centrifugation, culture on LJ media, and incubation at 37°C up to 8 weeks. The slants were inspected weekly. All positive cultures based on the WHO culture grading scale were confirmed for presence of AFB by ZN microscopy. Strain identification was done using temperature growth range, pigment production, resistance to p-nitrobenzoic acid (500 mg/L), and thiopen carboxylic acid hydrazide (2 mg/L) and niacin production [20].

Patients with at least one positive smear result (≥1 AFB/length) regardless of microscopy method were started on treatment. Patients without any positive smear were referred to the clinician for clinical and radiological investigation. Patients who were identified as culture-positive and not already on TB treatment were traced with the intention of offering treatment.

To ensure the quality of the NaOCl solution, 250 mL of bleach “working solution” was aliquoted weekly for processing specimens. Any remaining working solution was discarded at the end of the week. Before use, the presence of free chlorine was assessed using a swimming pool tester DPD1 (Pocket type Chlorine, range 0.1–6.0 mg/L) after prior dilution at d = 2.10^4^. In addition, monthly samples from the source NaOCl solution were sent to the biochemistry laboratory in KEMRI for iodometric titration of NaOCl.

### Sample size and statistical analysis

A sample size of 231 patients was calculated to detect at least 5% difference in sensitivity for TB detection between LED-FM on NaOCl-treated specimens and conventional ZN microscopy, with a proportion of 10% of discordant pairs and a power of 5%, using the McNemar's one-side test of equality of paired proportions (nQuery Advisor® 5.0) [6]. A minimum of 93 culture-positive patients were calculated to be required to measure minimum sensitivity of 60% for conventional ZN microscopy, with a precision of 10% and risk α of 5% (Epi Info 6.04, CDC, Atlanta, GA, USA). Using an estimation of 20% culture-positive patients among TB suspects, the sample size was increased to 510 patients.

Data were double entered using Epidata 3.1 (EpiData Association, Odense, Denmark) database and analyzed with Stata® 9.2 software (Stata Corp., College Station, TX, USA). Sensitivity, specificity, and positive and negative likelihood ratio for case detection were measured for all microscopy methods (direct ZN, LED-FM, and LED-FM after NaOCl sedimentation), using two different smear-positive case definitions: one positive smear (detection of at least 1 AFB per length) out of either 2 (most recent WHO smear-positive case definition) or 3 collected specimens. Sensitivity and specificity of LED-FM after NaOCl sedimentation were compared with those for direct ZN and direct LED-FM, using McNemar's test for matched data, respectively. Specimen and patients' detection yield of different methods were also compared.

A receiver operating characteristics (ROC) analysis was performed to define the best AFB cut-off to define a positive smear to be used with the LED-FM after NaOCl sedimentation when using the most recent WHO case definitions based on the collection of two specimens [24]. Inter-reader reliability of direct LED-FM and LED-FM after NaOCl sedimentation was assessed at smear level by calculating the Kappa coefficient (k). A Kappa between 0.80 and 1 signifies an almost perfect agreement. Mean times to stain batches of auramine slides and to individually read auramine smears were compared between direct LED-FM and LED-FM after NaOCl sedimentation with a paired t-test.

Proportion of treated patients and median time between first consultation and TB treatment initiation were measured for both smear-positive and smear-negative patients.

### Ethics

The Ethical Review Committee of KEMRI and the Comité de Protection des Personnes (Iles de France XI, Saint Germain en Laye, France) approved the study.

## Results

**Figure 1:**
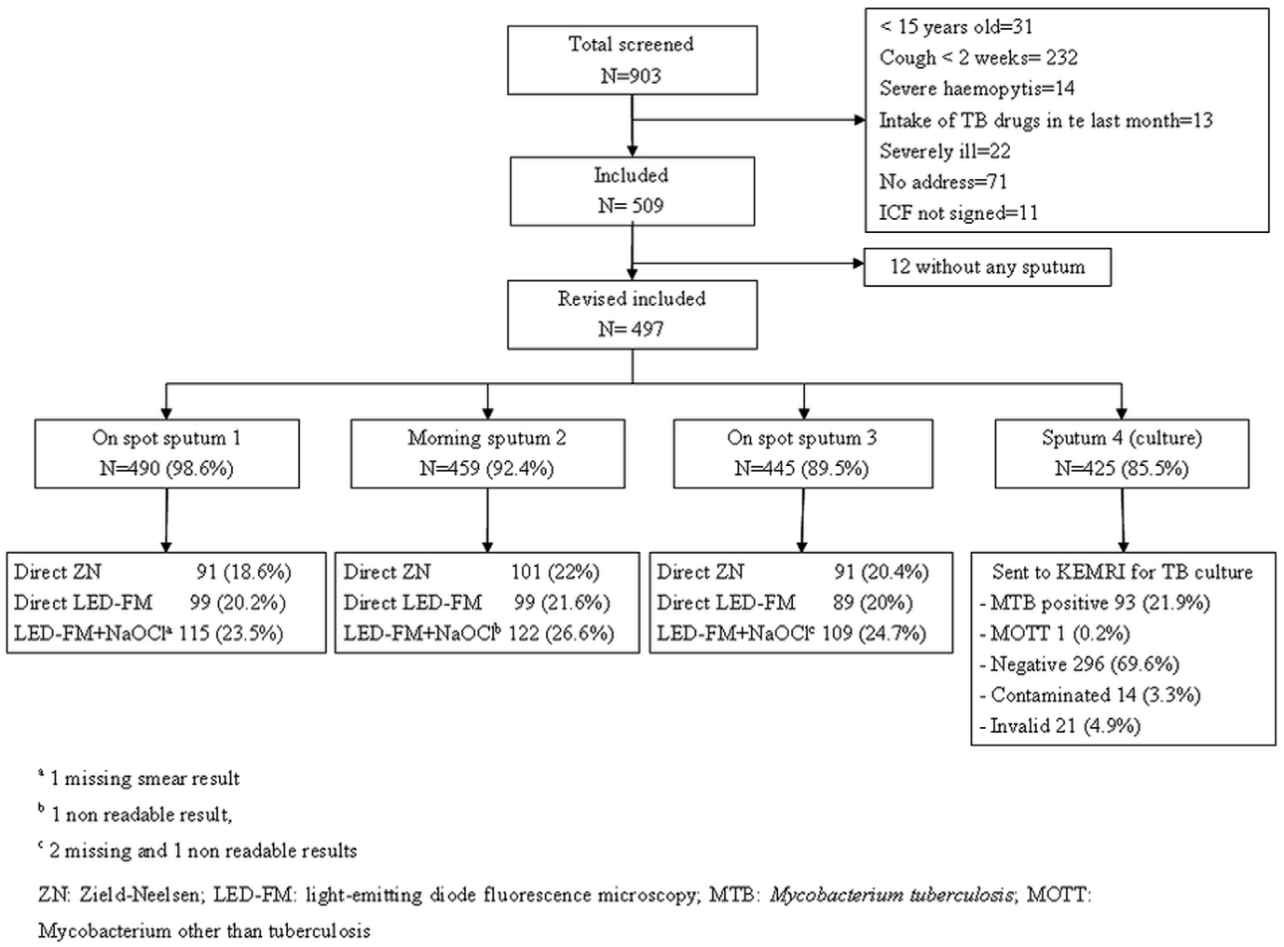
Study profile.

Between May 2008 and May 2009, a total of 509 pulmonary TB-suspected patients were included in the study (Figure 1). Male:female ratio was 1.6 (311∶198), and mean age (standard deviation, SD) was 34.3 years (SD 11.6). Patient characteristics were previously reported [20].

A total of 497 patients produced at least one specimen for smear microscopy, resulting in the collection of 1394 specimens. More than 80% of specimens were macroscopically of acceptable quality (purulent or mucopurulent) [20]. Detection yield of positive smears with LED-FM after NaOCl sedimentation was significantly higher than that with direct LED-FM (*P*<0.01) or direct ZN (*P*<0.01) methods (Table 1). The proportional incremental yield of LED-FM after NaOCl sedimentation over direct ZN and direct LED-FM was 22.3% (63/283) and 20.6% (59/287), respectively. The proportion of positive smears using LED-FM after NaOCl sedimentation was significantly higher among acceptable-quality smears (324/1255, 25.8%) compared to smears reported with background fluorescence or debris (22/130, 16.9%; *P* = 0.02).

**Table 1:** Smear microscopy results and smear microscopic appearance per microscopy methods.

	Direct ZN N = 1394	Direct LED-FM N = 1394	LED-FM after NaOCl N = 1391^2^
	n (%)	n (%)	n (%)
**≥1 AFB/length** 1	283 (20.3)	287 (20.6)	346 (24.9)
– Scanty	65 (23.0)	89 (31.0)	155 (44.2)
– 1+	101 (35.7)	61 (21.2)	46 (13.1)
– 2+	77 (27.2)	85 (29.6)	63 (17.9)
– 3+	40 (14.1)	52 (18.1)	82 (23.4)
**Non readable smears**	0	0	2 (0.1)
**Aspect of smear**			
– Good	1383 (99.2)	1371 (98.5)	1255 (90.2)
– Too tick	0	0	0
– Too thin	11 (0.8)	0	6 (0.4)
– Background-fluorescent	0	7 (0.5)	36 (2.6)
– Debris	0	16 (1.1)	94 (6.7)

NA  =  non applicable.

1 1 length = 100 HPF 1000× magnification (ZN) and 200 HPF under 400× magnification (FM).

2 3 slides broken.

ZN: Ziehl Neelsen; LED-FM: light-emitting diode fluorescence microscopy.

Using the smear-positive case definition of at least one positive smear out of 3 collected specimens, LED-FM after NaOCl sedimentation detected 151 smear-positive patients (30.4%, 95% CI: 26.4–34.6) compared to 115 (23.1%, 95% CI: 19.5–27.1) using direct ZN (*P*<0.01) and 113 (22.7%, 95% CI: 19.1–26.7) using direct LED-FM (*P*<0.01). This gave the LED-FM after NaOCl sedimentation method proportional incremental yields of 31% (36/115) and 34% (38/113), respectively, over direct LED-FM and direct ZN methods.

Using the most recent WHO smear-positive case definition of at least one positive smear out of 2 collected specimens (first on spot, second next morning), LED-FM after NaOCl sedimentation detected 141 smear-positive patients (28.4%, 95% CI: 24.4–32.5%) compared to 111 (22.3%, 95% CI: 18.7–26.5) with direct ZN (*P*<0.01) or direct LED-FM (*P*<0.01). The proportional incremental yield of LED-FM after NaOCl sedimentation over both direct LED-FM and direct ZN was 27% (30/111).

Seventy-two patients did not bring a fourth specimen for MTB culture (Figure 1). Of these, 55% were purulent or mucopurulent [20]. The smear positivity rate (≥1 positive smear out of 3 collected specimens) using ZN microscopy was not different between patients who produced a fourth specimen (97/425, 22.8%) and those who did not (16/72, 22.2%; *P* = 0.69). There were 21 invalid culture results due to laboratory errors in the specimen decontamination process. After exclusion of contaminated, invalid cultures and mycobacteria other than TB, 23.9% (93/389) of patients showed a positive culture of *Mycobacterium tuberculosis*.

Assessing performance of the different microscopy methods (Table 2) against the culture reference standard (ie, MTB culture) using a smear-positive case definition of one positive smear out of 3 collected specimens, no difference in sensitivity (*P* = 0.3) or specificity (*P* = 0.5) were found between direct LED-FM and direct ZN microscopy. LED-FM after NaOCl sedimentation appeared to be slightly more sensitive than direct LED-FM (*P* = 0.06) and direct ZN (*P* = 0.06), but this was not statistically significant.

**Table 2:** Performance (per patient) of different microscopy methods using smear-positive case definition of one positive smear out of three collected specimens.

	Direct ZN		Direct LED-FM		LED-FM after NaOCl	
	Pos	Neg	Pos	Neg	Pos	Neg
Positive culture	67	26	68	25	73	20
Negative culture	12	284	9	287	36	260
– Sensitivity, % [95%CI]	72.0 [61.8–81.6]		73.2 [62.9–81.8]		78.5 [68.8–86.3]	
– Specificity, % [95%CI]	95.9 [93.0–97.9]		96.7 [94.3–98.6]		87.8 [83.6–91.3]	
– Positive LR	17.8		24.0		6.45	
– Negative LR	0.29		0.28		0.24	

ZN: Ziehl Neelsen; LED-FM: light-emitting diode fluorescence microscopy; Pos: positive; Neg: negative.

Specificity of LED-FM after NaOCl sedimentation was significantly lower compared to direct LED-FM (*P*<0.001) and direct ZN (*P*<0.001). Indeed, LED-FM after NaOCl sedimentation resulted in 36 false smear-positive patients (corresponding to 59 smears). The majority of smears were scanty (98.3%) with a median AFB count of 1 (IQR 1–3). The presence of debris was reported in 5/59 (8.5%) of false-positive smears versus 9/192 (4.7%) true-positive smears (*P* = 0.6). Of the 59 false-positive specimens, 14 (23.7%) were also positive with direct ZN or/and direct LED-FM. Fifty-one LED-FM smears were re-stained and blindly read by the laboratory supervisor at the end of the study. Thirteen (25.5%) were confirmed as smear-positive.

**Figure 2:**
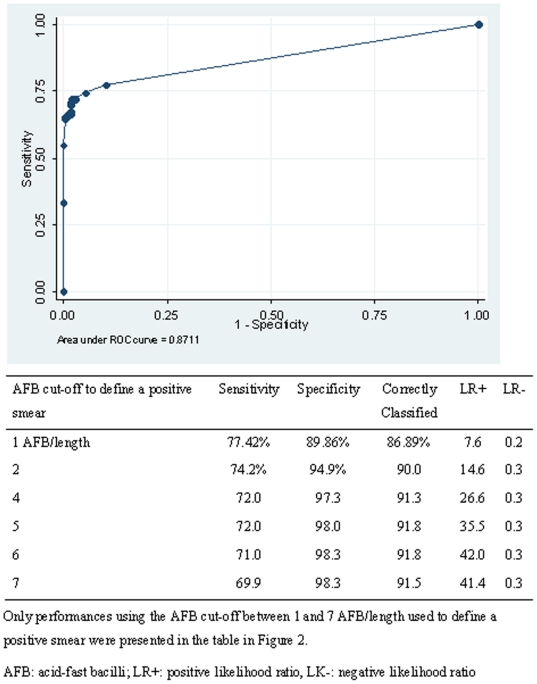
Performance of the LED-FM after NaOCl sedimentation using a case definition of one positive smear out of two collected specimens and different AFB cut-offs to define a positive smear.

When using the latest WHO smear-positive case definition (one positive smear out of 2 specimens), sensitivity of LED-FM after NaOCl sedimentation was 77.4% (95% CI: 67.6–95.4) compared to 72.0% (95% CI: 61.8–80.6) with direct LED-FM (*P* = 0.06) and 69.9% (95% CI: 59.5–78.0) with ZN (*P* = 0.03). Specificity of LED-FM after NaOCl sedimentation was 89.9% (95% CI: 85.8–93.0) compared to 97.3% (95% CI: 94.7–98.3) for direct LED-FM (*P*<0.01) and 96.6% (95% CI: 93.9–98.4) for ZN (*P*<0.01). Figure 2 presents the results of the ROC analysis of LED-FM performances after NaOCl sedimentation using case definition of at least one positive smear out of 2 collected specimens and different AFB cut-off to define a positive smear.

Inter-reader reliability of LED-FM after NaOCl sedimentation was favorable (k = 0.70, 95% CI: 0.70–0.87) but lower than for direct LED-FM (k = 0.90, 95% CI: 0.83–0.97). In internal and external ZN quality controls, 3/110 (2.7%) and 1/120 (0.8%) minor errors were detected, respectively [22].

Regarding field-program operational aspects, a median of 6 specimens (IQR 4–9) were processed per day. Mean duration of the staining procedure (drying, fixing, staining, and drying) was 30.7 min (SD 5.3) for LED-FM after NaOCl sedimentation (N = 346) compared to 28.5 min (SD 4.6) for direct LED-FM (N = 287; *P*<0.01). Mean duration of smear reading was 1.11 min (SD 0.38) for direct LED-FM and 1.34 min (SD 0.5) for LED-FM after NaOCl sedimentation (*P*<0.01). Monthly iodometric titration to assess bleach stability over the 1-year study period ranged between 3.5% and 4.5%. The results of the pool tester system used in the clinic on a weekly basis were consistent with the titration results done in KEMRI. In the ease-of-use questionnaire, technicians did not report any difficulty in reading smears after specimen processing, especially in focusing, compared to direct LED-FM method. Two results were invalid due to the smear washing off the slide.

Regarding treatment, of 160 smear-positive patients regardless of microscopy method, 150 (93.7%) were started on treatment, 9 (5.6%) defaulted before starting treatment, and one died (0.6%). Median time between the first consultation and treatment initiation was 3 days (IQR 2–6). Among the 337 remaining smear-negative patients, 51 (15.1%) were started on treatment. Forty-two (82.3%) of these 51 patients were started on treatment based on clinical and radiological findings with median delay of 5 days (IQR 4–11). Among them, 5 patients (11.9%) were confirmed culture-positive. Nine of 337 (17.6%) patients were started on treatment after culture results with a median time of 67 days (IQR 56–73) between first consultation and treatment initiation. Among the 18 smear-negative and culture-positive patients, 5 were already started on treatment before culture results, 9 were started after culture results, 2 could not be found, and 2 died before tracing.

## Discussion

This is the first study that evaluated the combination of LED-FM and overnight bleach sedimentation to improve TB case detection in patients at lower levels of health care services. NaOCl specimen sedimentation increased LED-FM detection yield by approximately 30% regardless of smear-positive case definition. The majority of additional positive smears were low scanty results. This increased yield was close to the 23% increase earlier reported in the same setting using identical processing methods combined with ZN microscopy [21]. However, when using MTB culture as reference standard, the difference in sensitivity in detecting cases between LED-FM after NaOCl sedimentation (78.5%) and direct LED-FM (73.2%) or direct ZN (72%) remained low.

Furthermore, LED-FM after NaOCl sedimentation was almost 10% less specific than the two direct methods. Patients identified with false smear-positive results had very low scanty results. Previous studies have shown that the probability of being culture-positive is reduced to 50% for specimens with smear results less than 3 AFB/300 fields [25]. One fourth of false smear-positive results had at least one extra positive smear result with one of the direct microscopy methods, and the same percentage was confirmed as smear-positive after an independent smear reading at the end of the study. These patients might have been wrongly classified as false positive and therefore the specificity was probably underestimated. Indeed, it is possible that mycobacteria from paucibacillary specimens were killed during the decontamination step resulting in false-negative culture results.

Also, the solid Lowenstein Jensen culture method used in this study is known to be 10–15% less sensitive than liquid culture methods [26]. The use of the Lowenstein Jensen culture and the storage of the culture specimen for 3–4 days on site before sending to KEMRI could potentially explain why some of the very low scanty specimens did not grow. This loss of specificity after NaOCl specimen sedimentation has been reported in another study in Ethiopia using ZN microscopy [27]. The authors attributed the decrease of specificity to the storage of specimens before culture, intensity of decontamination, and low amounts of specimens processed for culture. As shown in the ROC analysis, a threshold of 1 AFB/length to define a positive smear might be too low when using LED-FM after NaOCl specimen sedimentation. Indeed, using 2 instead of 1 AFB/length significantly increased specificity from 90% to 95%.

This study also addressed several field-program operational aspects of the bleach sedimentation method: 1) Deterioration of the bleach could be prevented using simple storage conditions in dark containers and cool areas, as shown by favorable results of monthly monitoring of iodometric titration [28]; 2) Use of a simple pool-tester system after dilution of bleach gave consistent results with iodometric titration and could be easier to use under program conditions; 3) With experienced technicians, the risk of false-negative results due to smears washing off slides during staining could be prevented, with only 2 cases out of 1394 smears; 4) Median duration of LED-FM smear reading was longer after bleach sedimentation compared to direct method but remained 3 times faster than the direct ZN method [21]; and 5) Difficulties in microscopy focusing earlier reported when the NaOCl sedimentation method was used in combination with ZN microscopy, due to lack of background material in the smears, was not reported by our staff when used in combination with LED-FM [21].

This study has several limitations. Because it was not possible to offer HIV testing and counseling to all TB suspects, the analysis could not be stratified per HIV status. For methodological reasons, ie, amount of specimen available, the culture reference standard was performed on a different specimen than those used for the microscopy methods. The macroscopic appearance of specimens used for culture was of poorer quality than specimens used for smear microscopy. Therefore, cultured specimens could have possibly contained fewer bacilli than microscopy specimens. This could have potentially biased the reported performance of the methods but is unlikely to have affected the difference in performance between direct microscopy and LED-FM after NaOCl sedimentation. Furthermore, not all patients were able to produce a fourth specimen for culture, although we do not believe this affected the overall results.

In conclusion, the use of combined NaOCl sedimentation and LED-FM in this study led to a significant increase in the yield of TB-positive smears compared to direct LED-FM. However with solid culture as the reference standard, no significant increase in sensitivity was observed. A significant reduction in specificity was seen, which could be explained by the increase of very low smear-positive scanty results when using LED-FM after NaOCl sedimentation [25]. Based on these results, we conclude that NaOCL sedimentation combined with LED-FM offers no major advantages over direct LED-FM, delays results, and is most likely a less accurate diagnostic method.
